# Biocontrol Agents Inhibit Banana Fusarium Wilt and Alter the Rooted Soil Bacterial Community in the Field

**DOI:** 10.3390/jof10110771

**Published:** 2024-11-06

**Authors:** Chanjuan Du, Di Yang, Shangbo Jiang, Jin Zhang, Yunfeng Ye, Lianfu Pan, Gang Fu

**Affiliations:** 1 Plant Protection Research Institute, Guangxi Academy of Agricultural Sciences/Guangxi Key Laboratory of Biology for Crop Diseases and Insect Pests/Key Laboratory of Green Prevention and Control on Fruits and Vegetables in South China Ministry of Agriculture and Rural Affairs, Nanning 530007, China; duchanjuan@gxaas.net (C.D.); dyang@gxaas.net (D.Y.); shbojiang@gxaas.net (S.J.); zhangjin0529@gxaas.net (J.Z.); panlianfu@gxaas.net (L.P.); 2 Horticultural Research Institute, Guangxi Academy of Agricultural Sciences, Nanning 530007, China; yeyunfeng@gxaas.net

**Keywords:** *Fusarium oxysporum* f. sp. *cubense*, biological control, rhizosphere microbiome

## Abstract

Banana is an important fruit and food crop in tropical and subtropical regions worldwide. Banana production is seriously threatened by Fusarium wilt of banana (FWB), a disease caused by *Fusarium oxysporum* f. sp. *cubense*, and biological control is an important means of curbing this soil-borne disease. To reveal the effects of biocontrol agents on inhibiting FWB and altering the soil bacterial community under natural ecosystems, we conducted experiments at a banana plantation. The control efficiency of a compound microbial agent (CM), *Paenibacillus polymyxa* (PP), *Trichoderma harzianum* (TH), and carbendazim (CA) on this disease were compared in the field. Meanwhile, the alterations in structure and function of the rooted soil bacterial community in different treatments during the vigorous growth and fruit development stages of banana were analyzed by microbiomics method. The results confirmed that the different biocontrol agents could effectively control FWB. In particular, CM significantly reduced the incidence of the disease and showed a field control efficiency of 60.53%. In terms of bacterial community, there were no significant differences in the richness and diversity of banana rooted soil bacteria among the different treatments at either growth stage, but their relative abundances differed substantially. CM treatment significantly increased the ratios of *Bacillus*, *Bryobacter*, *Pseudomonas*, *Jatrophihabitans*, *Hathewaya*, and *Chujaibacter* in the vigorous growth stage and *Jatrophihabitans*, *Occallatibacter*, *Cupriavidus*, and 1921-3 in the fruit development stage. Furthermore, bacterial community function in the banana rooted soil was affected differently by the various biocontrol agents. CM application increased the relative abundance of multiple soil bacterial functions, including carbohydrate metabolism, xenobiotic biodegradation and metabolism, terpenoid and polyketide metabolism, lipid metabolism, and metabolism of other amino acids. In summary, our results suggest that the tested biocontrol agents can effectively inhibit the occurrence of banana Fusarium wilt and alter the soil bacterial community in the field. They mainly modified the relative abundance of bacterial taxa and the metabolic functions rather than the richness and diversity. These findings provide a scientific basis for the use of biocontrol agents to control banana Fusarium wilt under field conditions, which serves as a reference for the study of the soil microbiological mechanisms of other biocontrol agents.

## 1. Introduction

Banana (*Musa* spp.) is one of the most important fruit and food crops in tropical and subtropical regions worldwide [1]. Fusarium wilt of banana (FWB), caused by *Fusarium oxysporum* f. sp. *cubense* (*Foc*) [2], is the most serious soil-borne fungal disease affecting the global banana industry [3,4]. The pathogen invades through the roots and spreads to the corm and pseudostem, eventually leading to plant wilting and death [5,6,7].

The main methods of control include the breeding of disease-resistant cultivars [8], chemical control [9,10], and biological control [11]. However, it is difficult to use conventional cross-breeding methods because the major banana cultivars are triploid and seedless. Therefore, there are still no resistant varieties suitable for commercial banana production [12]. In addition, the pathogen is difficult to kill using chemical fungicides owing to its strong ability to survive as viable chlamydospores in the soil, and we currently lack effective chemical fungicides for disease control [13]. Moreover, the long-term application of chemical fungicides pollutes the environment, damages soil physicochemical properties and microbial diversity, and leads to pathogen resistance [14]. As a sustainable and environmentally friendly control method, biological control has shown great potential for the prevention and control of soil-borne diseases [15,16]. Several biocontrol microorganisms, including *Bacillus* sp., *Pseudomonas* sp., *Burkholderia* sp., *Streptomyces* sp., *Trichoderma* sp., and *Rhizobium* sp., have been identified and used for FWB control. These strains have shown good biocontrol activity under laboratory and greenhouse conditions, but most have not been applied in the field [17,18,19,20]. In the previous study, we screened several biocontrol strains with high antagonistic effects against *Foc* from banana rhizosphere soil and developed a compound microbial agent composed of *Paenibacillus terrae*, *Burkholderia cepacia*, *Bacillus amyloliquefaciens*, and *Trichoderma harzianum* [21]. This compound microbial agent has shown stable, significantly higher control efficiency against Fusarium wilt of banana than single strains under field conditions.

The soil environment is a complex ecosystem, rich in microorganisms. The structure and composition of the soil microbial community are not only important for the maintenance of plant health and a stable soil microenvironment but are also closely related to various biological and chemical processes that inhibit soil-borne diseases [22,23,24]. The application of biocontrol agents in the field, which artificially introduces a large number of specific microbial species into natural soil, will certainly have consequences for the indigenous microbial community. Thus, understanding the effects of biocontrol agents on soil microbial community structure and function in field applications is important for further improving their control efficiency on banana Fusarium wilt.

Microbiome research techniques based on high-throughput sequencing have developed rapidly in recent years, providing new tools for in-depth analysis of the complex soil microbial communities of banana [25,26]. Zhou et al. [27] and Fatin et al. [28] reported that the abundance of *Foc* and the richness and diversity of fungi or bacteria were higher in the rhizosphere soil of banana infected with FWB than in that of healthy banana. The application of *B. amyloliquefaciens* to bananas planted in pots under greenhouse conditions caused changes in the rhizosphere microbial community, and the abundance of *Bacillus* and *Pseudomonas* increased significantly [29]. Likewise, Shen et al. [30] found that bio-organic fertilizer containing *Bacillus* sp. modified the structure of the soil bacterial community and effectively suppressed FWB. The abundance of *Gemmaticomonas* and *Sphingomonas* increased significantly, whereas that of *Bacteroidetes* decreased. Tao et al. [31] tracked the influence of a bio-organic fertilizer containing *B. amyloliquefaciens* W19 on the bulk and rhizosphere soil microbiome of banana and found an increase in the abundance of *Pseudomonas*. A bio-organic fertilizer containing *Trichoderma* was applied to control FWB in a pot experiment, resulting in a greater abundance of *Humicola* and a lower abundance of *Fusarium oxysporum* [32]. Although these studies characterized the rhizosphere soil microbial community of banana from various perspectives, most studies compared differences in soil microbial communities between healthy and infected bananas at a specific time point, evaluated the effect of biocontrol microorganisms on rhizosphere microbial communities of bananas in pots, or examined changes in microbial communities of the banana rhizosphere after applying bio-organic fertilizers in the field. Overall, most of the studies on FWB biocontrol have ignored the dynamic effects of biocontrol microbes on soil microbial communities in the field during different growth stages of banana. Due to the complexity of the field environment, much remains to be learned and clarified regarding the effects of biocontrol microbes on microbial community structure in banana rooted soil.

Here, we performed a field trial to assess the efficacy of biocontrol agents for controlling banana Fusarium wilt and examined the effects of different biocontrol agents on the composition and diversity of the rooted soil bacterial community at different plant developmental stages.

## 2. Materials and Methods

### 2.1. Biological Agents and Fungicides

The compound microbial agent was prepared by mixing equal volumes of liquid fermentation cultures of *Paenibacillus terrae*, *Burkholderia cepacia*, *Bacillus amyloliquefaciens*, and *Trichoderma harzianum* [21]. The effective concentration of viable cells was 1 × 10^9^ CFU·mL^−1^, and the mixture was provided by the Institute of Plant Protection, Guangxi Academy of Agricultural Sciences, Nanning, China.

*Paenibacillus polymyxa* (biocontrol agent, 5 × 10^9^ CFU·mL^−1^) was purchased from Wuhan Kernel Bio-tech Co., Ltd. (Wuhan, China). *T. harzianum* (biocontrol agent, 1 × 10^9^ spore·g^−1^) was purchased from Moon (Guangzhou) Biotech Co., Ltd. (Guangzhou, China). Carbendazim 50% wettable powder was purchased from Shanghai Yuelian Chemical Co., Ltd. (Shanghai, China).

### 2.2. Brief Information of Experiment Area

The field trial was conducted on a banana plantation in Tanluo Town, Nanning City, Guangxi Province, China (N 22°54′40″, E 107°54′49″). This area has a subtropical humid monsoon climate, with an average annual temperature of 21.6 °C, an average annual rainfall of 1048 mm, and an elevation of approximately 93 m above sea level. The soil type is red soil, and its physicochemical properties are as follows: pH 4.2, 28.7 g/kg soil organic matter, 131 mg/kg alkali-hydrolysable nitrogen, 47.5 mg/kg available phosphorus, and 301 mg/kg available potassium.

### 2.3. Field Experimental Design and Treatment

The overall size of the study area was 0.31 hectares, with 2.3 m interrow spacing and 2.0 m in-row spacing to give a planting density of 1875 plants·ha^−1^. The experimental site was a flat field that experienced severe Fusarium wilt of banana with an incidence rate of 22.7% and had been under continuous banana cultivation for 5 years. The tested plantlets were perennial banana (*Musa acuminata* L., AAA group, Cavendish subgroup, cv. Williams) with 8 to 10 true leaves. Field treatments were as follows: (1) compound microbial agent (CM) applied at 62.5 L·ha^−1^; (2) *P. polymyxa* (PP) applied at 12.5 L·ha^−1^; (3) *T. harzianum* (TH) applied at 62.5 kg·ha^−1^; (4) carbendazim (CA) applied at a rate of 420 g·ha^−1^; and (5) the same volume of water without biological agents or fungicides (control, CK). The field trial used a completely randomized block design with three replications for each treatment, and each treatment included 30 plants. The side rows at the edge served as protective rows.

**Figure 1:**
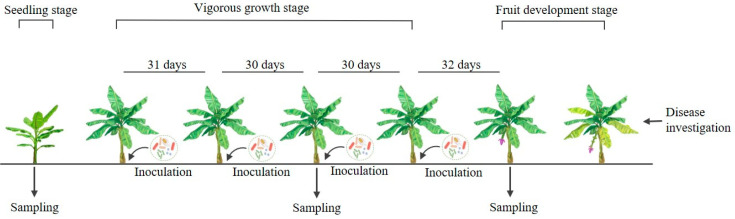
Experimental design of the field trial. The banana plant with yellow leaves shows symptoms of banana wilt. Samples were collected at the seedling, vigorous growth, and fruit development stages as shown. Biocontrol agent treatments were applied approximately every 30 days during the vigorous growth stage, for a total of four treatments.

The field trial was conducted from 30 April to 2 November 2022, as described in Fu et al. [33] and Damodaran et al. [34], with slight modifications. Treatments were applied during the vigorous growth stage of banana. The first inoculation was performed on 2 June 2022, with 0.35 L of inoculum applied around the roots of each plant. The treatments were then applied three more times, approximately every 30 days (Figure 1). Cultivation and management conditions were consistent throughout the experiment. During the fruit development stage, the numbers of infected plants in each treatment were counted on 2 November, and control efficiency (%) was calculated according to the following formula (*n* = 30) [35]:

$$\mathrm{Control}\;\mathrm{efficiency}\;(\%)=\frac{\left(N1-N2\right)}{N1}\times 100$$

N_1_, number of infected plants in the control area; N_2_, number of infected plants in the treatment area.

### 2.4. Soil Sample Collection

Bulk soil samples near the plant roots were collected (more than 1 cm from the root surface) using a 50 mm soil auger at the seedling stage (before treatment application, on 30 April 2022), the vigorous growth stage (30 days after the second treatment, on 2 August 2022), and the fruit development stage (32 days after the fourth treatment, on 2 October 2022) (Figure 1). Bulk soil samples were randomly collected from 5 points in an “S” shape in each experimental subplot. The depth of sample collection was 20 cm, and the distance between two adjacent points was not less than 10 m. Soil samples collected from each plot were mixed after large pieces of debris were removed and were then homogenized and passed through a 2 mm sieve. All soil samples were placed in 50 mL sterile centrifuge tubes and stored at −80 °C for DNA extraction.

### 2.5. DNA Extraction and Amplicon Sequencing

Total DNA was extracted from soil using the HiPure Soil DNA Kit (Magen, Guangzhou, China) according to the manufacturer’s protocols, and the quantity and concentration of total DNA were determined using a NanoDrop ND-2000 instrument (Thermo Fisher Scientific, Wilmington, DE, USA). Amplicons of the V3–V4 hypervariable region of the *16S rRNA* gene were obtained using the universal primers 341F (5′-CCTACGGGNGGCWGCAG-3′)/806R (5′-GGACTACHVGGGTATCTAAT-3′). The amplification reaction was performed as follows: 95 °C pre-denaturation for 5 min; 30 cycles of denaturation at 95 °C for 60 s, annealing at 60 °C for 60 s; extension at 72 °C for 60 s; and a final extension at 72 °C for 7 min. PCR amplicons were extracted from 2% (*w*/*v*) agarose gels and purified using the AxyPrep DNA Gel Extraction Kit (Axygen Biosciences, Union City, CA, USA), then quantified using the ABI StepOnePlus Real-Time PCR System (Life Technologies, Foster City, CA, USA). The purified amplicons were used to construct libraries, which were sequenced on the Novaseq 6000 platform (Illumina, San Diego, CA, USA) according to the standard protocols of Gene Denovo Biotechnology Co., Ltd. (Guangzhou, China).

### 2.6. Analysis of Sequence Data

Raw reads were filtered using FASTP (version 0.18.0): (1) remove reads containing more than 10% of unknown nucleotides, (2) remove reads containing less than 50% of bases with quality (Q-value) > 20, and (3) remove adapter contamination [36]. The clean reads were then assembled into tags using FLASH (version 1.2.11) [37]. The effective tags were clustered into operational taxonomic units (OTUs) with ≥97% similarity using the UPARSE (version 9.2.64) pipeline [38]. The representative sequence (the most abundant tag sequence in each OTU) was used to classify OTUs into taxa by a naive Bayesian model using an RDP classifier based on the SILVA database [39], with a confidence threshold value of 0.8.

OTU alpha diversity metrics, including the Shannon, Simpson, Chao1, and ACE indices, were calculated in QIIME (version 1.9.1) to examine the richness and diversity of the bacterial community in different samples [40]. Rarefaction curves were plotted using the ggplot2 R package (version 2.2.1) [41]. Using OTU expression data, principal coordinate analysis (PCoA) based on weighted UniFrac distance was performed with the Vegan R package (version 2.5.3) [42], and the results were plotted with ggplot2 (version 2.2.1). The abundance statistics for each taxon were visualized using Krona (version 2.6) [43], and a stacked bar plot of community composition was created using ggplot2 (version 2.2.1). To identify unique and common species, Venn diagrams were created with the VennDiagram R package (version 1.6.16) [44] and upset plots with the UpSetR package (version 1.3.3) [45]. The linear discriminant analysis (LDA) effect size (LEfSe) method was used to identify taxa whose abundance differed significantly between treatments and could therefore serve as biomarkers [42]. Welch’s *t*-test was used to detect functions whose abundance differed significantly between pairs of treatments.

### 2.7. Statistical Analysis

The significance of differences among treatments was assessed by one-way analysis of variance (one-way ANOVA), with a significance level of *p* < 0.05 using SPSS Statistics 18.0 (IBM, New York, NY, USA).

## 3. Results

### 3.1. Field Control Efficiency of Biocontrol Agents for Banana Fusarium Wilt

**Figure 2:**
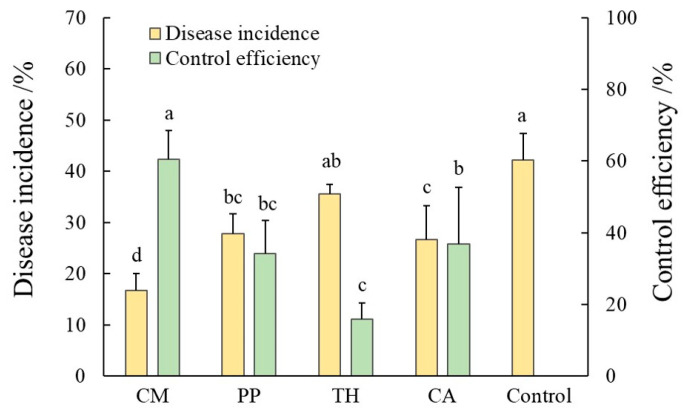
Effects of different inoculation treatments on the disease incidence and control efficiency of banana Fusarium wilt. CM, compound microbial agent; PP, *Paenibacillus polymyxa*; TH, *Trichoderma harzianum*; CA, carbendazim. Significant differences (*p* < 0.05) among treatments are indicated by different letters.

We evaluated the control efficiencies of different biocontrol agents for banana Fusarium wilt in the field and compared them to that of the chemical fungicide carbendazim (Figure 2). After four consecutive applications during the vigorous growth stage, the disease incidence was significantly lower in the CM, PP, and CA treatments than in the control treatment (*p* < 0.05). The disease incidence of the control treatment was 42.22%, whereas those of the CM, PP, and CA treatments were 16.67%, 27.78%, and 26.67%, respectively. Notably, disease incidence was significantly lower in the CM treatment than in the carbendazim treatment. All treatments showed some degree of control efficiency against FWB, and significant differences are indicated in Figure 2 (*p* < 0.05). The CM treatment showed the highest control efficiency at 60.53%, and the TH treatment showed the lowest control efficiency at 15.79% (*p* < 0.05). There were no significant differences in control efficiency among the TH, PP, and CA treatments. Thus, the CM treatment was most effective in controlling banana Fusarium wilt.

### 3.2. Richness and Diversity of the Bacterial Community in Banana Rooted Soil in the Field

Samples of banana rooted soil were collected for high-throughput sequencing of the V3–V4 region of the *16S rRNA* gene on the Illumina Novaseq 6000 platform. A total of 4,171,528 raw tags were detected from the 33 samples. After quality control and chimera removal, 3,547,109 valid sequences were retained for all samples. Their lengths ranged from 201 to 474 bp, with a mean of 451 bp. The average Good’s coverage exceeded 98.26% for all samples, indicating that the sequencing accuracy was reliable. The high-quality sequences were clustered into operational taxonomic units (OTUs) based on 97% sequence similarity, and 3812 OTUs were obtained. These OTUs were annotated to 25 phyla, 68 classes, 136 orders, 205 families, 286 genera, and 11 species. Rarefaction curves based on the Sobs index tended to be flat, indicating that the sequencing depth was sufficient to cover most of the bacterial species in the banana rooted soil.

**Figure 3:**
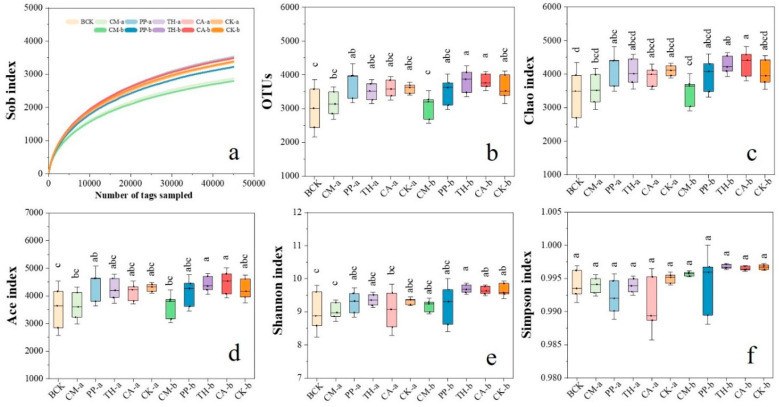
Bacterial richness and diversity of banana rooted soils at the seedling, vigorous growth, and fruit development stages. (**a**) Sob rarefaction curve; (**b**) Number of operational taxonomic units (OTUs); (**c**) Chao index; (**d**) Ace index; (**e**) Shannon index; (**f**) Simpson index. CM, compound microbial agent; PP, *Paenibacillus polymyxa*; TH, *Trichoderma harzianum*; CA, carbendazim; BCK, before treatment; CK, control. Letters a and b indicate the vigorous growth and fruit development stages, respectively. Values are the means ± standard deviations (SD) (*n* = 3). Different letters indicate significant differences among the treatments (*p* < 0.05).

The richness and diversity metrics of the soil bacterial community remained relatively stable throughout the entire growth period (Figure 3). Alpha diversity indices were calculated, and there were no significant differences in the Chao index (Figure 3b), Ace index (Figure 3c), or number of OTUs (Figure 3d) between the CM, PP, TH, and CA treatments and the control at any growth stage (*p* < 0.05), indicating that these four treatments did not alter the richness of the bacterial community. Likewise, there were no significant differences in the Shannon (Figure 3e) or Simpson (Figure 3f) indices between the CM, PP, TH, and CA treatments and the control at any growth stage (*p* < 0.05), indicating that these four treatments did not alter the diversity of the bacterial community. Overall, the application of CM did not affect the richness or diversity of bacteria in banana rooted soils in the field.

### 3.3. Relative Abundance of the Bacterial Community in Banana Rooted Soil in the Field

**Figure 4:**
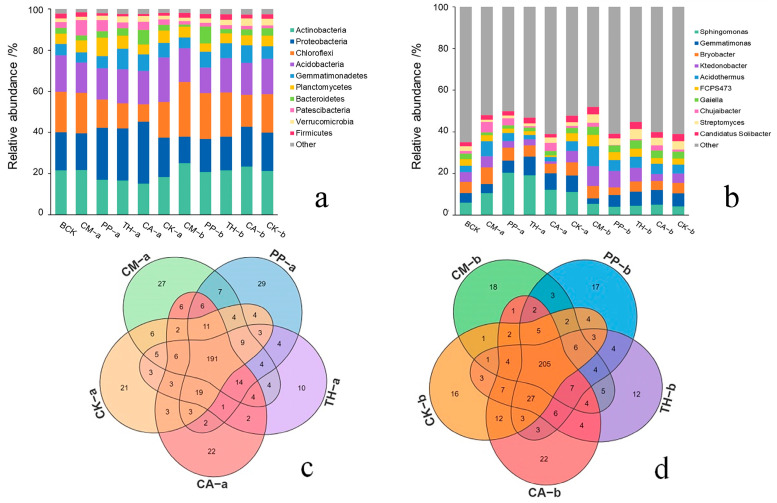
Bacterial composition of banana rooted soil from different treatments. (**a**,**b**) Bacterial composition at the phylum level (**a**) and genus level (**b**). (**c**,**d**) Venn diagrams showing shared and unique bacterial genera in the five treatments at the vigorous growth stage (**c**) and fruit development stage (**d**). CM, compound microbial agent; PP, *Paenibacillus polymyxa*; TH, *Trichoderma harzianum*; CA, carbendazim; BCK, before treatment; CK, control. Letters a and b indicate the vigorous growth and fruit development stages, respectively. “Other” indicates the relative abundance of all phylum-level or genus-level classifications other than the top 10 presented and named in the lists (**a**,**b**).

Relative abundance reflects the proportion of bacterial taxa in the overall community. Using the species annotation results, we created bar graphs showing differences in the relative abundance of major microbial taxa at the phylum (Figure 4a) and genus (Figure 4b) levels in banana rooted soils. The relative abundance of various bacterial taxa differed between the treatment groups and the control.

At the phylum level (Figure 4a), *Actinobacteria*, *Proteobacteria*, *Chloroflexi*, *Acidobacteria*, *Gemmatimonadetes*, *Planctomycetes*, *Bacteroidetes*, *Patescibacteria*, *Verrucomicrobia*, and *Firmicutes* were the top 10 bacterial phyla in banana rooted soil in the field. Compared with other treatments, the CM treatment showed a higher relative abundance of *Actinobacteria*, *Chloroflexi*, *Patescibacteria*, and *Firmicutes* at the vigorous growth stage and of *Actinobacteria*, *Chloroflexi*, and *Planctomycetes* at the fruit development stage. CM application decreased the relative abundance of *Proteobacteria*, *Acidobacteria*, *Gemmatimonadetes*, *Bacteroidetes*, and *Verrucomicrobia* at both stages. Notably, the relative abundance of *Acidobacteria* was consistently higher in the control treatment than in the biocontrol treatments.

At the genus level (Figure 4b), *Sphingomonas*, *Gemmatimonas*, *Bryobacter*, *Ktedonobacter*, *Acidothermus*, FCPS473, *Gaiella*, *Chujaibacter*, *Streptomyces*, and *Candidatus Solibacter* were the top 10 bacterial genera in banana rooted soil. Compared with the other treatments, the CM treatment showed a higher relative abundance of *Bryobacter*, *Acidothermus*, and *Chujaibacter* and a lower relative abundance of *Sphingomonas*, *Gemmatimonas*, *Gaiella*, and *Streptomyces* at the vigorous growth stage; likewise, the CM treatment showed a higher relative abundance of *Sphingomonas*, *Bryobacter*, *Acidothermus*, and FCPS473 and a lower relative abundance of *Gemmatimonas* at the fruit development stage. Notably, the relative abundance of *Sphingomonas* gradually decreased in banana rooted soil over the course of the field trial and was higher in the CM treatment than in the other treatments at the fruit development stage.

To visualize differences in soil bacterial composition among treatments, we generated a Venn diagram showing the numbers of shared and unique genera in each treatment. Common and unique bacterial genera varied greatly among the different treatments at the same growth stage (Figure 4c,d). At the vigorous growth stage, 191 bacterial genera were common to all treatments, whereas 27 were unique to CM, 29 to PP, 10 to TH, 22 to CA, and 21 to the control (Figure 4c). At the fruit development stage, 205 bacterial genera were common to all treatments, whereas 18 were unique to CM, 17 to PP, 12 to TH, 22 to CA, and 16 to the control (Figure 4d).

### 3.4. Principal Coordinate Analysis of Bacterial Communities in Banana Rooted Soil

**Figure 5:**
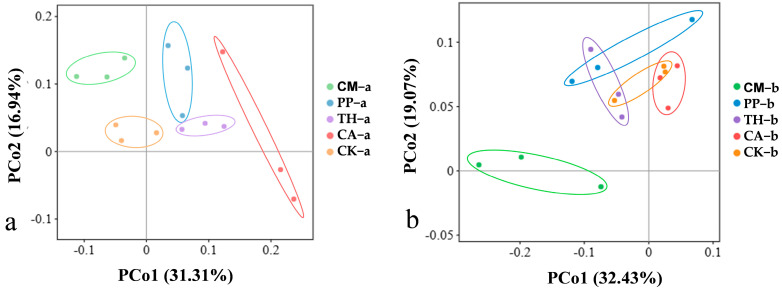
Weighted UniFrac principal coordinate analysis (PCoA) of the soil bacterial communities of banana under different treatments. (**a**) Vigorous growth stage; (**b**) Fruit development stage. CM, compound microbial agent; PP, *Paenibacillus polymyxa*; TH, *Trichoderma harzianum*; CA, carbendazim; CK, control. Letters a and b indicate the vigorous growth and fruit development stages, respectively.

To investigate the effects of different biocontrol agents on bacterial community structure in banana rooted soil, we performed principal coordinate analysis (PCoA) of all samples at the OTU level. In a PCoA plot based on weighted UniFrac distance (Figure 5), different samples from the same treatment tended to cluster together (circled areas). At the vigorous growth stage (Figure 5a), the first two PCoA axes explained 48.25% of the total variance in soil bacterial community structure (PCo1, 31.31%; PCo2, 16.94%). All treatment groups were clearly separated from the control group, indicating that the bacterial community structure changed markedly after the application of different biocontrol agents. At the fruit development stage (Figure 5b), the first two PCoA axes explained 51.50% of the total variance in soil bacterial community structure (PCo1, 32.43%; PCo2 19.07%). The PP, TH, and CA treatment groups were closer to the control group, indicating that their bacterial community structures were similar; by contrast, the CM group was clearly separated from all other groups. In general, the application of CM had a strong effect on bacterial community structure in banana rooted soil in the field.

### 3.5. Significant Differences in the Abundance of Bacterial Taxa in Banana Rooted Soils

**Figure 6:**
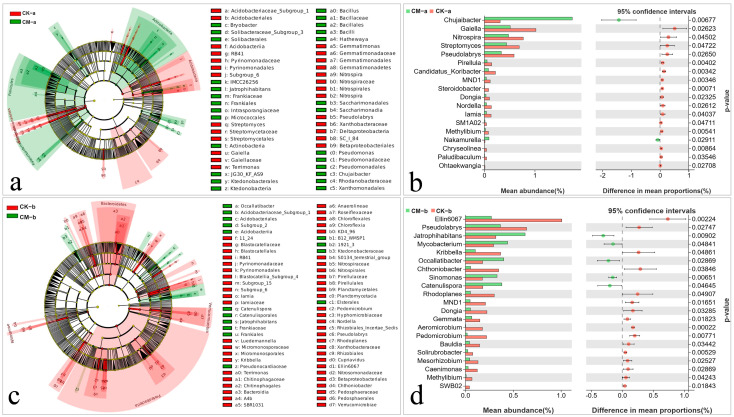
Differences in the abundance of bacterial taxa and identification of biomarkers associated with the compound microbial agent (CM) and control (CK) treatments. (**a**,**b**) LEfSe analysis of the bacterial community (**a**) and Welch’s *t*-tests of bacterial abundance at the genus level (**b**) at the vigorous growth stage. (**c**,**d**) LEfSe analysis of the bacterial community (**c**) and Welch’s *t*-tests of bacterial abundance at the genus level (**d**) at the fruit development stage. In (**a**,**c**), tracks from inside to outside represent classification levels from phylum to genus. Each small solid circle represents a taxon at that level, and the size of the circle is proportional to the relative abundance of the taxon. Taxa with an LDA score > 3.0 and *p* < 0.05 are shown. In (**b**,**d**), *n* = 3 and *p* < 0.05.

To identify possible biomarkers, we performed LEfSe analysis to examine significant differences in the relative abundance of bacterial taxa between the CM and control treatments. Fifty-eight taxa whose relative abundance differed significantly between the CM and control treatments were identified at the vigorous growth stage (LDA score > 3.0, *p* < 0.05) (Figure 6a). The relative abundance of *Bacillus*, *Bryobacter*, *Pseudomonas*, *Jatrophihabitans*, *Hathewaya*, and *Chujaibacter* was significantly higher in the CM treatment, whereas *Streptomyces*, *Nitrospira*, *Gaiella*, *Pseudolabrys*, *Terrimonas*, RB41, and *Gemmatimonas* were enriched in the control treatment. Sixty-eight taxa with significant differences in abundance were found at the fruit development stage (LDA score > 3.0, *p* < 0.05) (Figure 6c). The relative abundance of *Jatrophihabitans*, *Occallatibacter*, *Cupriavidus*, and 1921-3 (unspecified bacterial genera belonging to *Chloroflexi*, *Ketedonobacteria*, *Ktedonobacterales*, *Ktedonobacteraceae*) was significantly higher in the CM treatment, whereas *Rhodoplanes*, *Nordella*, *Iamia*, *Pseudolabrys*, *Terrimonas*, RB41, *Luedemannella*, *Kribbella*, Ellin6067, *Pedomicrobium*, *Cupriavidus*, and *Chthoniobacter* were enriched in the control treatment.

Differences in bacterial communities between the CM and control treatments were also analyzed by Welch’s *t*-test. Compared with the control treatment, the CM treatment had significantly higher relative abundance of *Chujaibacter* and *Nakamurella* in the vigorous growth stage and of *Jatrophihabitans*, *Mycobacterium*, *Occallatibacter*, *Sinomonas*, and *Catenulispora* in the fruit development stage in the field.

### 3.6. Prediction of Bacterial Community Function in Banana Rooted Soils

**Figure 7:**
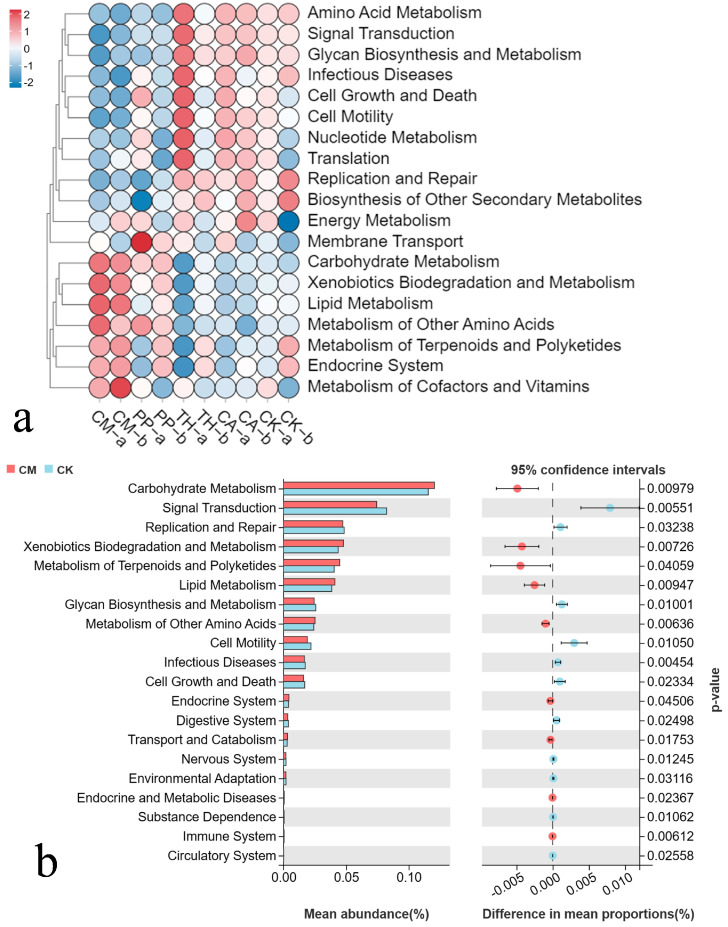
Metabolic functions of bacterial communities of banana rooted soil predicted by Tax4Fun. (**a**) Clustered heatmap showing the relative abundance of the top 20 KEGG metabolic pathways across all samples; red indicates higher abundance and blue indicates lower abundance. CM, compound microbial agent; PP, *Paenibacillus polymyxa*; TH, *Trichoderma harzianum*; CA, carbendazim; CK, control. The letters a and b indicate the vigorous growth stage and fruit development stage, respectively. (**b**) Significant differences in the abundance of different functions between the CM and CK treatments were analyzed with Welch’s *t*-test (*n* = 3, *p* < 0.05).

To investigate the effects of different biocontrol agents on the functional capabilities of bacterial communities in banana rooted soils, we used Tax4Fun to predict the KEGG pathways of bacteria in all samples, and 37 different KEGG pathways were identified. On the basis of KEGG level-2, the top 20 functions and their abundance in each sample were used to construct a heat map. Clustering analysis was performed according to differences and similarities in functions among the samples. As shown in Figure 7a, the various biocontrol agents had different effects on bacterial community function in banana rooted soil. The CM and PP treatments formed one cluster, and the TH, CA, and control treatments formed another, suggesting that the community functions of soil bacteria were more similar in the CM and PP treatments and in the TH, CA, and control treatments. From these results, we speculated that different biocontrol agents induce various degrees of resistance to banana Fusarium wilt by regulating bacterial community functions in banana rooted soil.

Differences in the abundance of various functional annotations between the CM and control treatments were analyzed by Welch’s *t*-test. As shown in Figure 7b, upregulated KEGG pathways in the CM treatment were mainly associated with carbohydrate metabolism, xenobiotics biodegradation and metabolism, metabolism of terpenoids and polyketides, lipid metabolism, and metabolism of other amino acids (*p* < 0.05), whereas downregulated KEGG pathways were associated with signal transduction, replication and repair, glycan biosynthesis and metabolism, cell motility, infectious diseases, and cell growth and death (*p* < 0.05). By contrast, these downregulated KEGG pathways were enriched in the control treatment. Notably, the upregulated KEGG pathways in the CM treatment were consistent in the vigorous growth and fruit development stages (Figure 7a), indicating that the community structure and function of the soil bacteria in the field were relatively stable in the CM treatment compared with the other treatments.

## 4. Discussion

A number of studies have suggested that the application of biocontrol microorganisms to banana rhizosphere soil can significantly increase the richness and diversity of soil microorganisms, thus inhibiting the occurrence of banana Fusarium wilt [29,31,32,33,46,47]. In this study, we performed a field trial of biocontrol agents against FWB and investigated the effects of these agents on the bacterial community of banana rooted soil by high-throughput sequencing. Our results confirmed that the application of a compound microbial agent significantly inhibited the occurrence of the disease. In contrast to the studies mentioned above, we found that the application of the compound microbial agent did not change the bacterial richness or diversity of banana rooted soil (Figure 3), but it did significantly change bacterial community structure (Figure 4 and Figure 5). Because the components of the compound microbial agent were all derived from banana rhizosphere soil [21], its large-scale application in the field is less likely to change soil bacterial diversity. By contrast, biocontrol agents that are introduced artificially may engage in complex competitive or synergistic interactions with indigenous microorganisms in the field, which in turn may alter the original soil microbial community structure.

Soil microorganisms are an essential indicator of soil health [48]. Changes in soil microbial community composition are often related to disease inhibition [23,24]. In this study, the application of a compound microbial agent significantly increased the relative abundance of *Actinobacteria*, *Chloroflexi*, *Firmicutes*, *Planctomycetes*, *Bryobacter*, *Acidothermus*, and *Chujaibacter* in banana rooted soil and decreased that of *Proteobacteria*, *Acidobacteria*, *Gemmatimonadetes*, and *Bacteroidetes* (Figure 4a,b). LEfSe analysis indicated that *Bacillus*, *Bryobacter*, *Pseudomonas*, *Jatrophihabitans*, *Hathewaya*, and *Chujaibacter* (at the vigorous growth stage; Figure 6a,b) and *Jatrophihabitans*, *Occallatibacter*, *Cupriavidus*, and 1921-3 (at the fruit development stage; Figure 6c,d) may be biomarkers of bacterial communities that can control the occurrence of banana Fusarium wilt after the application of compound microbial agents.

*Actinobacteria* are involved in the decomposition of soil organic matter such as lignin and cellulose [49,50]. *Chloroflexi* are a class of photosynthetic autotrophic bacteria with a strong capacity for CO_2_ fixation and biological phosphorus removal [51], and some are involved in nitrite oxidation [52]. Members of *Firmicutes* include many important beneficial bacteria, such as *Bacillus*, that have shown inhibitory effects toward multiple fungal plant diseases [53]. *Planctomycetes* are a class of bacteria with anammox function involved in the global nitrogen cycle [54]. *Bryobacter* and *Acidothermus* can decompose lignin and cellulose, thus improving soil fertility [55]. These beneficial bacteria were highly enriched in CM-treated banana rooted soil, potentially helping to improve the unbalanced soil environment and thus playing an important role in inhibiting banana Fusarium wilt.

*Proteobacteria* are widely distributed in nature and include many pathogenic bacteria that cause plant diseases. Previous studies reported that the inhibition of cucumber and banana Fusarium wilt was associated with a lower relative abundance of *Proteobacteria* in soil [56,57]. Most species in *Acidobacteria* are acidophilic bacteria that act as parasites in acidic soils [58], and acidic soil environments can promote the occurrence of FWB [59]. Therefore, the relative abundance of *Acidobacteria* in soil may be positively associated with the incidence of FWB. Although *Gemmatimonadetes* and *Bacteroidetes* participate in the degradation of cellulose and participate in the soil N cycle and nutrient turnover [60,61], some are known to be opportunistic pathogens that release toxic substances during proteolysis [62]. The relative abundance of these harmful bacteria decreased upon CM application in banana rooted soil, suggesting that compound microbial agents may help to reduce soil acidity, leading to inhibition of banana Fusarium wilt.

Although the relative abundance of *Sphingomonas* gradually decreased in banana rooted soil as the banana plants grew and developed, it was higher in the CM treatment than in other treatments at the fruit development stage (Figure 4b). Root exudate composition can differ among growth stages, causing changes in soil microbial communities [63,64]. Changes in the relative abundance of *Sphingomonas* at different growth stages may therefore have been caused by changes in the root exudates of banana. In addition, *Sphingomonas* has shown inhibitory effects on plant pathogenic fungi [65] that can protect host plants from pathogens [66]. Here, the results of LEfSe analysis (Figure 6) showed that although *Sphingomonas* was not a biomarker associated with the occurrence of banana Fusarium wilt, its enrichment in banana rooted soil was induced by the application of a compound microbial agent during the fruit development stage (Figure 4b), potentially slowing or inhibiting the development of the disease.

When the bacterial community composition in the banana rooted soil changed, its metabolism and functional output changed accordingly. To obtain a deeper understanding of the links between bacterial community structure and function in banana rooted soil, we predicted the metabolic functions of the bacterial community using Tax4Fun. Carbohydrate metabolism, xenobiotics biodegradation and metabolism, metabolism of terpenoids and polyketides, lipid metabolism, and metabolism of other amino acids were upregulated in the CM treatment relative to the control treatment. *Firmicutes* has been reported as a dominant member of soil bacterial communities and can improve the soil environment by increasing carbohydrate metabolism and regulating lipid metabolism [67]. *Firmicutes* has also been suggested to have a degree of bactericide resistance and strong biodegradation ability, showing bioremediation capabilities on acidified or heavy-metal-contaminated soils [68]. *Actinobacteria* are significant producers of bioactive secondary metabolites with various biological activities. These secondary metabolites include polyketides, terpenoids, and peptides and have shown antibacterial activity [69]. Biocontrol agents may enrich these metabolic pathways by regulating bacterial community structure in banana rooted soil, leading to higher resistance to Fusarium wilt of banana. Bacterial community functions enriched in the control treatment were mainly related to infectious diseases and cell growth and death (Figure 7b), perhaps explaining why there was a higher disease incidence in the control treatment.

The changes above suggest that the introduction of a large number of microorganisms into natural ecosystems can change the community structure of indigenous microorganisms, thereby changing the original function of the soil. As we known, some of the changes were beneficial, while some were harmful [70] (Patoine et al., 2022). These results further demonstrated that human activities can affect the composition of soil microbial communities and provide a valuable reference for scientific research in this area.

## 5. Conclusions

Our results suggest that the tested biocontrol agents can effectively inhibit the occurrence of banana Fusarium wilt and alter the soil bacterial community in the field. They mainly modified the relative abundance of bacterial taxa and the metabolic functions rather than the richness and diversity. Compared with other treatments, the application of compound microbial agents significantly reduced the incidence of banana Fusarium wilt, with a control efficiency of 60.53%, and significantly increased the ratios of beneficial bacteria, including *Bacillus*, *Bryobacter*, *Pseudomonas*, and *Jatrophihabitans* in banana rooted soil. CM application increased the relative abundance of multiple soil bacterial functions, including carbohydrate metabolism, xenobiotic biodegradation and metabolism, terpenoid and polyketide metabolism, lipid metabolism, and metabolism of other amino acids. These findings provide a scientific basis for the use of biocontrol agents in controlling banana. These findings provide a scientific basis for the use of biocontrol agents in controlling banana Fusarium wilt under field conditions, which serve as a reference for the study of soil microbiological mechanisms of other biocontrol agents.

## Data Availability

Data will be made available on request.

## References

[1] Scott G.J. (2021). A review of root, tuber and banana crops in developing countries: Past, present, and future. Int. J. Food Sci. Technol..

[2] Nel B., Steinberg C., Labuschagne N., Viljoen A. (2006). Isolation and characterization of nonpathogenic *Fusarium oxysporum* isolates from the rhizosphere of healthy banana plants. Plant Pathol..

[3] Butler D. (2013). Fungus threatens top banana. Nature.

[4] Li C.Y., Mostert G., Zuo C.W., Beukes I., Yang Q.S., Sheng O., Kuang R.B., Wei Y.R., Hu C.H., Rose L. (2013). Diversity and distribution of the banana wilt pathogen *Fusarium oxysporum* f. sp. cubense in China. Fungal Genom. Biol..

[5] Saravanan T., Muthusamy M., Marimuthu T. (2003). Development of integrated approach to manage the fusarial wilt of banana. Crop Prot..

[6] Ploetz R.C., Jones D. (2019). Fusarium wilt. Handbook of Diseases of Banana, Abacá and Ense.

[7] Pegg K.G., Coates L.M., O’Neill W.T., Turner D.W. (2019). The epidemiology of Fusarium wilt of banana. Front. Plant Sci..

[8] Ploetz R.C. (2015). Management of Fusarium wilt of banana: A review with special reference to tropical race 4. Crop Prot..

[9] Nel B., Steinberg C., Labuschagne N., Viljoen A. (2007). Evaluation of fungicides and sterilants for potential application in the management of Fusarium wilt of banana. Crop Prot..

[10] Nguyen T.V., Tran-Nguygen L.T.T., Wright C.L., Trevorrow P., Grice K. (2019). Evaluation of the efficacy of commercial disinfectants against *Fusarium oxysporum* f. sp. *cubense* race 1 and tropical race 4 propagules. Plant Dis..

[11] Zhang H., Mallik A., Zeng R.S. (2013). Control of Panama disease of banana by rotating and intercropping with Chinese chive (Allium Tuberosum Rottler): Role of plant volatiles. J. Chem. Ecol..

[12] Wang X.Y., Yu R.B., Li J.Y. (2021). Using genetic engineering techniques to develop banana cultivars with Fusarium wilt resistance and ideal plant architecture. Front. Plant Sci..

[13] Ghag S.B., Shekhawat U.K.S., Ganapathi T.R. (2015). Fusarium wilt of banana: Biology, epidemiology and management. Int. J. Pest Manag..

[14] Mandal A., Sarkar B., Mandal S., Vithanage M., Patra A.K., Manna M.C. (2020). Impact of agrochemicals on soil health. Agrochem. Detect. Treat. Remediat..

[15] Fravel D., Olivain C., Alabouvette C. (2003). *Fusarium oxysporum* and its biocontrol. New Phytol..

[16] Lahlali R., Ezrari S., Radouane N., Kenfaoui J., Esmaeel Q., Hamss H.E., Belabess Z., Barka E.A. (2022). Biological control of plant pathogens: A global perspective. Microorganisms.

[17] Fishal E.M.M., Meon S., Yun W.M. (2010). Induction of tolerance to Fusarium wilt and defense-related mechanisms in the plantlets of susceptible Berangan banana pre-inoculated with *Pseudomonas* sp. (UPMP3) and *Burkholderia* sp. (UPMB3). Agric. Sci. China.

[18] Sekhar A.C., Thomas P. (2015). Isolation and identification of shoot-tip associated endophytic bacteria from banana cv. Grand Naine and testing for antagonistic activity against *Fusarium oxysporum* f. sp. *cubense*. Am. J. Plant Sci..

[19] Raza W., Ling N., Zhang R.F., Huang Q.W., Xu Y.C., Shen Q.R. (2017). Success evaluation of the biological control of Fusarium wilts of cucumber, banana, and tomato since 2000 and future research strategies. Crit. Rev. Biotechnol..

[20] Wang J., Cai B.Y., Li K., Zhao Y.K., Li C.Y., Liu S.W., Xiang D.D., Zhang L., Xie J.H., Wang W. (2022). Biological control of *Fusarium oxysporum* f. sp. *cubense* tropical race 4 in banana plantlets using newly isolated *Streptomyces* sp. WHL7 from marine soft coral. Plant Dis..

[21] Du C.J., Yang D., Ye Y.F., Pan L.F., Zhang J., Jiang S.B., Fu G. (2022). Construction of a compound microbial agent for biocontrol against Fusarium wilt of banana. Front. Microbiol..

[22] Mueller U.G., Sachs J.L. (2015). Engineering microbiomes to improve plant and animal health. Trends Microbiol..

[23] Raaijmakers J.M., Mazzola M. (2016). ECOLOGY. Soil immune responses. Science.

[24] Kwak M.J., Kong H.G., Choi K., Kwon S.K., Song J.Y., Lee J., Lee P.A., Choi S.Y., Seo M., Lee H.J. (2018). Rhizosphere microbiome structure alters to enable wilt resistance in tomato. Nat. Biotechnol..

[25] Metzker M.L. (2010). Sequencing technologies-the next generation. Nat. Rev. Genet..

[26] Pollock J., Glendinning L., Wisedchanwet T., Watson M. (2018). The madness of microbiome: Attempting to find consensus “best practice” for 16S microbiome studies. Appl. Environ. Microbiol..

[27] Zhou D.B., Jing T., Chen Y.F., Wang F., Qi D.F., Feng R.J., Xie J.H., Li H.P. (2019). Deciphering microbial diversity associated with Fusarium wilt-diseased and disease-free banana rhizosphere soil. BMC Microbiol..

[28] Fatin N.J., Amalia M.H., Mohd T.Y., Noor B.S. (2022). Analysis of soil bacterial communities and physicochemical properties associated with Fusarium wilt disease of banana in Malaysia. Sci. Rep..

[29] Tian L.B., Zhang W.L., Zhou G.D., Li S., Wang Y.F., Yang B.M., Bai T.T., Fan H.C., He P., Zheng S.J. (2023). A biological product of *Bacillus amyloliquefaciens* QST713 strain for promoting banana plant growth and modifying rhizosphere soil microbial diversity and community composition. Front. Microbiol..

[30] Shen Z.Z., Wang D.S., Ruan Y.Z., Xue C., Zhang J., Li R., Shen Q.R. (2014). Deep 16S rRNA Pyrosequencing Reveals a Bacterial Community Associated with Banana Fusarium Wilt Disease Suppression Induced by Bio-Organic Fertilizer Application. PLoS ONE.

[31] Tao C.Y., Li R., Xiong W., Shen Z.Z., Liu S.S., Wang B.B., Ruan Y.Z., Geisen S., Shen Q.R., Kowalchuk G.A. (2020). Bio-organic fertilizers stimulate indigenous soil Pseudomonas populations to enhance plant disease suppression. Microbiome.

[32] Tao C.Y., Wang Z., Liu S.S., Lv N.N., Deng X.H., Xiong W., Shen Z.Z., Zhang N., Geisen S., Li R. (2023). Additive fungal interactions drive biocontrol of Fusarium wilt disease. New Phytol..

[33] Fu L., Ruan Y.Z., Tao C.Y., Li R., Shen Q.R. (2016). Continous application of bioorganic fertilizer induced resilient culturable bacteria community associated with banana Fusarium wilt suppression. Sci. Rep..

[34] Damodaran T., Rajan S., Muthukumar M., Gopal R., Yadav K., Kumar S., Ahmad I., Kumari N., Mishra V.K., Jha S.K. (2020). Biological management of banana Fusarium wilt caused by *Fusarium oxysporum* f. sp. *cubense* tropical race 4 using antagonistic fungal isolate CSR-T -3 (*Trichoderma reesei*). Front. Microbiol..

[35] Wang B.B., Shen Z.Z., Zhang F.G., Waseem R., Yuan J., Huang R., Ruan Y.Z., Li R., Shen Q.R. (2016). *Bacillus amyloliquefaciens* strain W19 can promote growth and yield and suppress Fusarium wilt in banana under greenhouse and field conditions. Pedosphere.

[36] Chen S.F., Zhou Y.Q., Chen Y.R., Gu J. (2018). fastp: An ultra-fast all-in-one FASTQ preprocessor. Bioinformatics.

[37] Magoč T., Salzberg S.L. (2011). FLASH: Fast length adjustment of short reads to improve genome assemblies. Bioinformatics.

[38] Edgar R.C. (2013). UPARSE: Highly accurate OTU sequences from microbial amplicon reads. Nat. Methods.

[39] Quast C., Pruesse E., Yilmaz P., Gerken J., Schweer T., Yarza P., Peplies J., Glöckner F.O. (2013). The SILVA ribosomal RNA gene database project: Improved data processing and web-based tools. Nucleic Acids Res..

[40] Caporaso J.G., Kuczynski J., Stombaugh J., Bittinger K., Bushman F.D., Costello E.K., Fierer N., Pena A.G., Goodrich J.K., Gordon J.I. (2010). QIIME allows analysis of high-throughput community sequencing data. Nat. Methods.

[41] Wickham H. (2011). ggplot2. Wiley Interdiscip. Rev. Comput. Stat..

[42] Segata N., Izard J., Waldron L., Gevers D., Miropolsky L., Garrett W.S., Huttenhower C. (2011). Metagenomic biomarker discovery and explanation. Genome Biol..

[43] Ondov B.D., Bergman N.H., Phillippy A.M. (2011). Interactive metagenomic visualization in a Web browser. BMC Bioinform..

[44] Chen H., Boutros P.C. (2011). VennDiagram: A package for the generation of highly-customizable Venn and Euler diagrams in R. BMC Bioinform..

[45] Conway J.R., Lex A., Gehlenborg N. (2017). UpSetR: An R package for the visualization of intersecting sets and their properties. Bioinformatics.

[46] Qiu M.H., Zhang R.F., Xue C., Zhang S.S., Li S.Q., Zhang N., Shen Q.R. (2012). Application of bio-organic fertilizer can control Fusarium wilt of cucumber plants by regulating microbial community of rhizosphere soil. Biol. Fertil. Soils.

[47] Shen Z.Z., Zhong S.T., Wang Y.G., Wang B.B., Mei X.L., Li R., Ruan Y.Z., Shen Q.R. (2013). Induced soil microbial suppression of banana fusarium wilt disease using compost and biofertilizers to improve yield and quality. Eur. J. Soil Biol..

[48] Yuan J., Wen T., Zhang H., Zhao M.L., Penton C.R., Thomashow L.S., Shen Q.R. (2020). Predicting disease occurrence with high accuracy based on soil macroecological patterns of Fusarium wilt. ISME J..

[49] Lechevalier H., Sykes G., Skinner F.A. (1973). Actinomycetales: Characteristics and Practical Importance: The Society for Applied Bacteriology Symposium Series No. 2.

[50] Lynd L.R., Weimer P.J., Van Zyl W.H., Pretorius I.S. (2022). Microbial cellulose utilization: Fundamentals and biotechnology. Microbiol. Mol. Biol. Rev..

[51] Xian W.D., Zhang X.T., Li W.J. (2020). Research status and prospect on bacterial phylum *Chloroflexi*. Acta Microbiol. Sin..

[52] Spieck E., Spohn M., Wendt K., Bock E., Shively J., Frank J., Indenbirken D., Alawi M., Lücker S., Hüpeden J. (2020). Extremophilic nitrite-oxidizing *Chloroflexi* from Yellowstone hot springs. ISME J..

[53] Saxena A.K., Kumar M., Chakdar H., Anuroopa N., Bagyaraj D.J. (2020). *Bacillus* species in soil as a natural resource for plant health and nutrition. J. Appl. Microbiol..

[54] Wiegand S., Jogler M., Boedeker C., Pinto D., Vollmers J., Rivas-Marín E., Kohn T., Peeters S.H., Heuer A., Rast P. (2020). Cultivation and functional characterization of 79 Planctomycetes uncovers their unique biology. Nat. Microbiol..

[55] Wang J., Gao G., Li Y.W., Yang L.Z., Liang Y.L., Jin H.Y., Han W.W., Feng Y., Zhang Z.M. (2015). Cloning, expression, and characterization of a thermophilic endoglucanase, AcCel12B from *Acidothermus cellulolyticus* 11B. Int. J. Mol. Sci..

[56] Yao H.Y., Wu F.Z. (2010). Soil microbial community structure in cucumber rhizosphere of different resistance cultivars to Fusarium wilt. FEMS Microbiol. Ecol..

[57] Shen Z.Z., Ruan Y., Xue C., Zhang J., Li R., Shen Q.R. (2015). Rhizosphere microbial community manipulated by 2 years of consecutive biofertilizer application associated with banana Fusarium wilt disease suppression. Biol. Fertil. Soils.

[58] Dedysh S.N., Yilmaz P. (2018). Refining the taxonomic structure of the phylum *Acidobacteria*. Int. J. Syst. Evol. Microbiol..

[59] Segura R.A., Stoorvogel J.J., Sandoval J.A. (2022). The effect of soil properties on the relation between soil management and fusarium wilt expression in Gros Michel bananas. Plant Soil.

[60] Lladó S., Zifcakova L., Vetrovsky T., Eichlerova I., Baldrian P. (2016). Functional screening of abundant bacteria from acidic forest soil indicates the metabolic potential of *Acidobacteria* subdivision 1 for polysaccharide decomposition. Biol. Fertil. Soils.

[61] Wegner C.E., Liesack W. (2016). Microbial community dynamics during the early stages of plant polymer breakdown in paddy soil. Environ. Microbiol..

[62] Johnson E.L., Heaver S.L., Walters W.A., Ley R.E. (2017). Microbiome and metabolic disease: Revisiting the bacterial phylum *Bacteroidetes*. J. Mol. Med..

[63] Chaparro J.M., Badri D.V., Vivanco J.M. (2014). Rhizosphere microbiome assemblage is affected by plant development. ISME J..

[64] Schlemper T.R., Leite M.F.A., Lucheta A.R., Shimels M., Bouwmeester H.J., Veen J.A.V., Kuramae E.E. (2017). Rhizobacterial community structure differences among *Sorghum* cultivars in different growth stages and soils. FEMS Microbiol. Ecol..

[65] Wachowska U., Irzykowski W., Jędryczka M., Stasiulewicz-Paluch A.D., Głowacka K. (2013). Biological control of winter wheat pathogens with the use of antagonistic *Sphingomonas* bacteria under greenhouse conditions. Biocontrol Sci. Technol..

[66] Innerebner G., Knief C., Vorholt J.A. (2011). Protection of Arabidopsis thaliana against leaf-pathogenic *Pseudomonas syringae* by *Sphingomonas* strains in a controlled model system. Appl. Environ. Microbiol..

[67] Kumar Singh A., Cabral C., Kumar R., Ganguly R., Kumar Rana H., Gupta A., Rosaria Lauro M., Carbone C., Reis F., Pandey A.K. (2019). Beneficial Effects of Dietary Polyphenols on Gut Microbiota and Strategies to Improve Delivery Efficiency. Nutrients.

[68] Li W.X., Zhang Y.P., Mao W., Wang C.S., Yin S.X. (2020). Functional potential differences between Firmicutes and Proteobacteria in response to manure amendment in a reclaimed soil. Can. J. Microbiol..

[69] Siddharth S., Rai V.R. (2019). Isolation and characterization of bioactive compounds with antibacterial, antioxidant and enzyme inhibitory activities from marine-derived rare actinobacteria, *Nocardiopsis* sp. SCA21. Microb. Pathog..

[70] Patoine G., Eisenhauer N., Cesarz S. (2022). Drivers and trends of global soil microbial carbon over two decades. Nat. Commun..

